# Supplementary figures and images for: The qualitative assessment of optical coherence tomography and the central retinal sensitivity in patients with retinitis pigmentosa
Source: PLoS One. 2020 May 11;15(5):e0232700. doi: 10.1371/journal.pone.0232700 (PMC7213731; doi:10.1371/journal.pone.0232700)

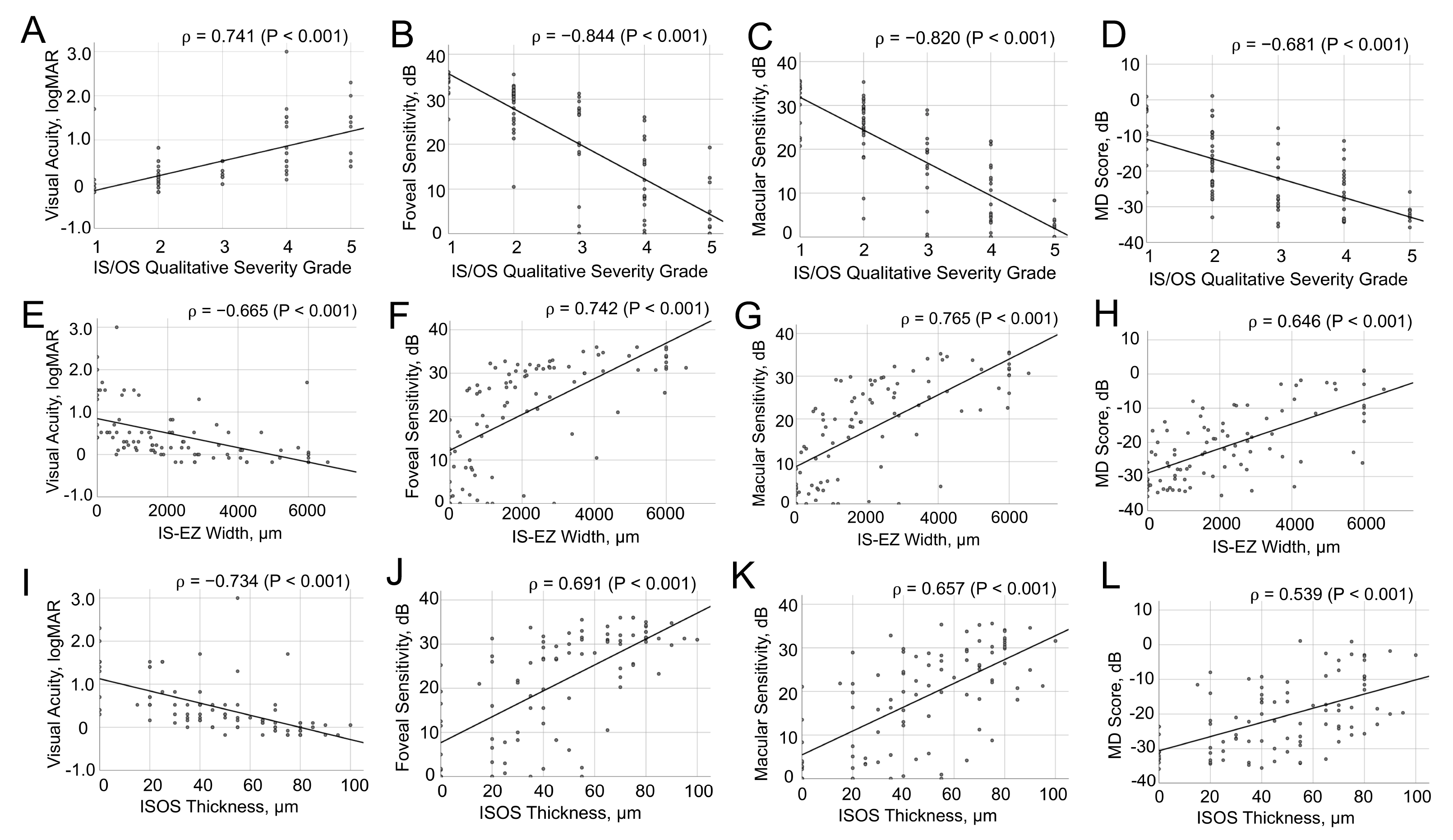

Supplement: S1 Fig — (TIF) [file pone.0232700.s001.tif]

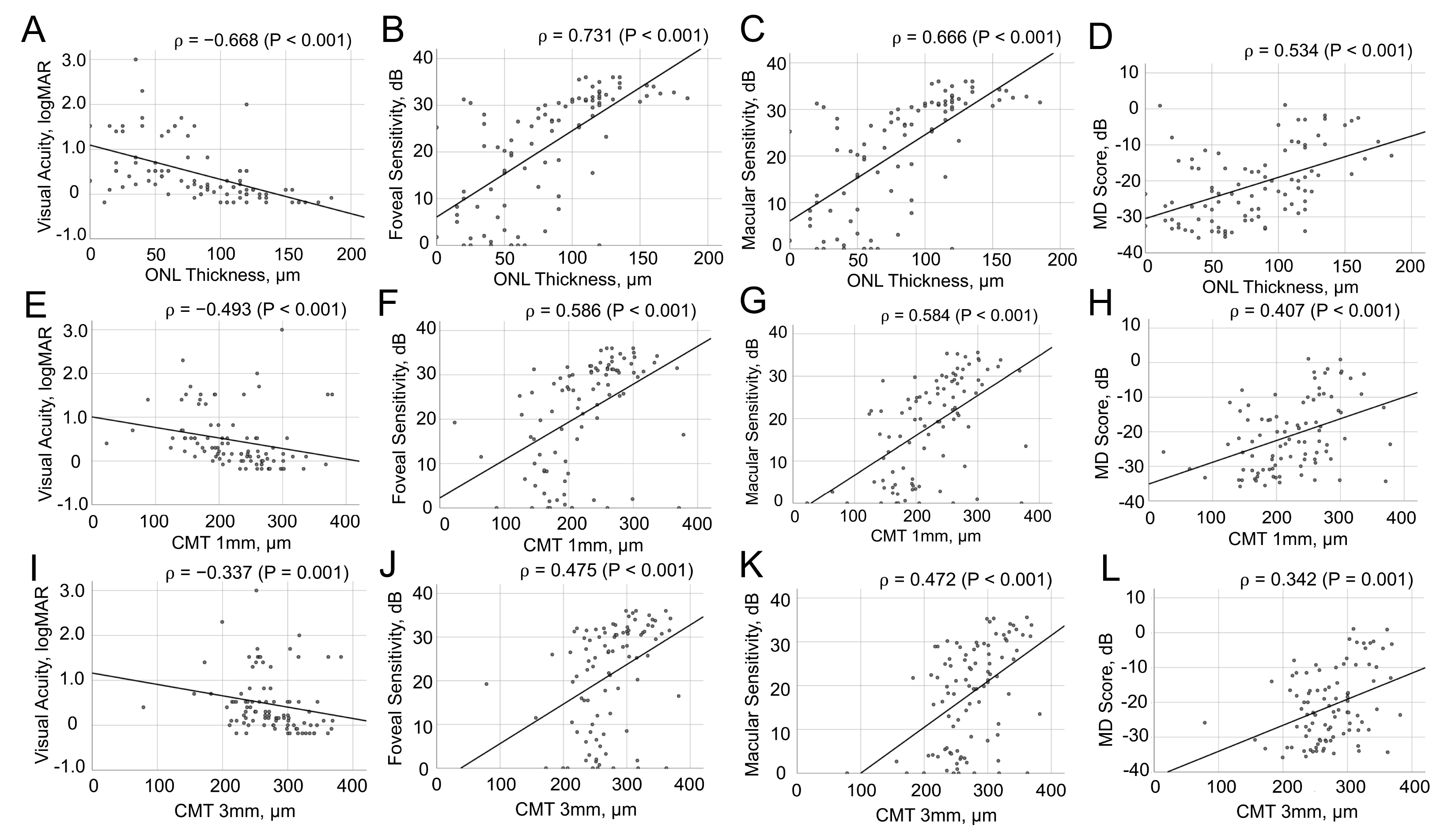

Supplement: S2 Fig — (TIF) [file pone.0232700.s002.tif]
