# Supplementary material for: The qualitative assessment of optical coherence tomography and the central retinal sensitivity in patients with retinitis pigmentosa
Source: PLoS One. 2020 May 11;15(5):e0232700. doi: 10.1371/journal.pone.0232700 (PMC7213731; doi:10.1371/journal.pone.0232700)
Supplement: S1 Table — (PDF) [file pone.0232700.s003.pdf]

Table S1: Raw Data of Quantitative Parameters of SD-OCT findings

Patient's No.	IS-OS thickness	IS-EZ width	ONL thickness	CMT 1mm	CMT 3mm	Cube volume	Cube thickness
1	20	480	35	143	182.3	6.3	174
2	65	4970	160	302	340.4	9.3	259
3	40	140	40	155	253.4	11.5	319
4	0	0	25	170	251.3	9	250
5	0	0	25	171	254.1	7.4	207
6	0	0	15	148	258	8.8	245
7	80	3300	185	368	367.1	10.9	303
8	0	0	55	205	206.6	7.6	210
9	30	550	20	147	245.7	6.3	175
10	35	880	50	146	232.7	8.4	233
11	55	1560	115	261	293.9	9.4	260
12	30	2430	70	174	271.6	9	250
13	90	1640	95	201	232.8	8.3	231
14	80	1530	120	249	282.1	9.5	264
15	0	0	75	179	273.9	9.8	271
16	30	590	15	162	221.6	7.6	212
17	40	280	115	213	238.6	9.3	258
18	25	0	80	193	240.8	7.9	218
19	55	2750	120	260	302.9	10.1	282
20	65	2400	110	274	294.2	7.1	196
21	70	910	50	244	316.9	8.6	240
22	100	6000	115	276	325.3	10.4	289
23	45	2130	115	307	345.7	8.8	244
24	40	1570	90	200	271.3	7.8	217
25	70	5220	150	316	355.1	10.7	296
26	75	5960	70	263	303.7	7.6	211
27	80	6550	100	260	327.8	10.5	292
28	0	750	0	124	250	7.7	213
29	70	1520	55	214	237.6	7.7	214
30	0	0	20	64	156.4	5.3	147
31	45	440	30	166	242.7	8.4	233
32	45	540	60	190	210.9	6.8	188
33	55	2850	120	267	278.3	6.4	177
34	0	0	40	143	199.4	7.1	196
35	55	2590	110	245	265.4	8.7	242
36	0	0	120	260	317.8	9.5	265
37	25	620	90	199	263.4	9.5	263
38	20	1260	20	146	217.1	6.6	184
39	0	220	65	378	381.8	11.8	327
40	0	0	55	23	78.6	4.2	116
41	50	6000	165	297	340.6	9.8	272
42	20	110	50	162	299.8	10.5	293
43	35	280	60	205	278.8	9.4	261
44	20	1040	65	371	362.3	10.1	280
45	80	4250	120	278	322.4	8.7	241
46	50	1170	45	194	235.6	7.5	207
47	20	694	15	132	252	9.6	266
48	25	760	40	194	256.7	9.5	263
49	20	3390	50	141	228.6	7.3	202
50	35	830	105	285	333	10.2	284

51	55	2890	55	168	246.2	8.6	240
52	65	4070	90	195	213.4	6.8	190
53	30	2140	35	222	273.6	7.8	218
5	55	550	35	299	251.9	9	251
55	35	6000	135	286	344.9	10.5	291
56	80	6000	115	267	313	9	251
57	20	350	50	162	166.9	8.7	241
58	85	2890	130	269	300.1	8.6	238
59	80	6000	105	242	236.4	9	250
60	65	5710	100	235	301.7	9.9	275
61	50	1680	35	177	265.4	9.6	266
62	75	5190	135	259	314.6	9.2	255
63	70	3740	80	206	280.4	9	249
64	15	4660	45	127	220.8	7.9	220
65	60	3450	80	246	300.2	9.3	257
66	70	1880	120	235	263.9	7.8	217
67	40	2050	60	182	235.6	7	195
68	90	4250	135	280	303.1	9	251
69	65	1830	90	235	282.1	9.4	261
70	65	1120	125	262	300.9	6.7	186
71	40	2460	115	198	251.1	8.3	229
72	80	3700	120	301	335.2	9.7	269
73	40	1470	55	156	214.7	7.9	220
74	20	1180	20	88	172.4	6.5	182
75	40	2080	75	187	241.7	7.3	204
76	95	2770	110	267	300.3	8.4	234
77	75	6000	110	300	361.6	10.7	298
78	40	2090	25	301	284.1	8.5	235
79	85	1590	125	244	246.7	7.7	214
80	50	1030	80	189	265.9	9.1	252
81	80	2770	175	333	333.7	8.9	247
82	75	2390	105	279	315.7	9.3	258
83	45	1810	85	238	277.8	9.7	269
84	20	760	75	198	235.8	7.1	199
85	35	750	90	220	286.2	8.1	224
86	75	2220	155	233	221.2	6	168
87	55	6000	105	250	309.3	9.9	276
88	70	2770	85	216	239.8	8.5	236
89	50	3730	120	258	256.4	8.2	228
90	0	80	0	239	316.6	8.2	227
91	70	4070	130	267	298.8	8.2	227
92	80	4120	155	337	368.8	10.2	283
93	40	1090	85	207	270.6	7.2	199

Abbreviations: IS-OS, photoreceptor inner and outer segments; IS-EZ, photoreceptr inner segment ellipsoid zone; ONL, outer nuclear layer; CMT, central retnal thickness
