# Supplementary material for: The qualitative assessment of optical coherence tomography and the central retinal sensitivity in patients with retinitis pigmentosa
Source: PLoS One. 2020 May 11;15(5):e0232700. doi: 10.1371/journal.pone.0232700 (PMC7213731; doi:10.1371/journal.pone.0232700)
Supplement: S2 Table — (PDF) [file pone.0232700.s004.pdf]

Table S2: Raw Data of the Central Retinal Sensitivities

Patient's No.	logMAR	FS	MS	MD
1	0.7	26	21.75	-14.29
2	-0.18	34	33.75	-2.52
3	1.7	12	12.17	-16.6
4	1.4	1.5	2.17	-33.12
5	1.52	0	0	-32.1
6	1.52	5	3.67	-31
7	-0.08	31.5	31.25	-12.64
8	0.4	0	4	-33.42
9	0.52	10	10.67	-26.41
10	0.22	15.5	5.42	-34.3
11	0.22	27.75	19.83	-29.46
12	0.3	21.75	15.83	-21.49
13	0.05	29.75	24.92	-20.12
14	0.1	32.75	29.83	-10.37
15	1.30	12.5	8.33	-31.34
16	0.10	8.25	3.75	-26.56
17	0.52	15.5	13	-20.36
18	1.52	3	4.67	-27.97
19	0.52	31.5	25	-28.13
20	0.16	31	29.58	-9.31
21	0.52	20.25	11.25	-28.17
22	0.05	31	31.58	-2.91
23	0.52	29.5	28	-22.34
24	0.22	26.75	24.08	-15.66
25	0.10	30.75	31.58	-4.53
26	1.70	25.5	22.58	-26.03
27	-0.18	31.25	30.67	-4.46
28	0.30	25.25	21.08	-23.64
29	0.40	22.5	18.25	-23.52
30	0.70	11.5	2.67	-30.76
31	0.40	8.25	4.42	-33.67
32	0.00	26.5	22.42	-16.54
33	0.00	33	28.25	-22.75
34	2.30	0	0	-35.83

35	0.16	31	27	-26.47
36	2.00	0	0	-33.98
37	0.82	7.75	3.42	-33.29
38	0.52	31.25	28.92	-7.97
39	1.52	16.5	13.5	-23.62
40	0.40	19.25	0	-25.85
41	-0.18	32.5	26	-13.88
42	0.52	8.5	7.42	-22.84
43	0.52	0	0	-34.68
44	1.52	0	0	-34.33
45	-0.18	30.5	26.42	-25.68
46	0.30	6	5.75	-27.01
47	1.40	6.5	5.08	-27.21
48	1.52	0.75	3.25	-30.7
49	0.70	16	16.33	-21.9
50	0.30	25.75	18.08	-27.88
51	1.30	0	0	-34.17
52	0.10	10.5	4.17	-32.98
53	0.82	21.25	23.75	-27.18
5	3.00	2	0.92	-34
55	0.00	33.75	32.83	-9.86
56	-0.08	34	31.83	-9.21
57	0.52	3.25	2.42	-31.21
58	0.00	31.25	20.75	-18.47
59	-0.18	31.5	30.17	-11.53
60	0.16	30.75	27.17	-22.93
61	0.22	28	21.33	-16.38
62	-0.08	36	22.08	-2.76
63	-0.08	30	25.08	-24.19
64	0.52	21	21.83	-11.54
65	0.22	28	23.17	-17.54
66	0.00	32	25.83	-18.99
67	0.52	1.75	0.58	-35.55
68	-0.18	34.75	34.58	-1.83
69	0.16	30.5	19.25	-18.93
70	0.10	32.25	29.17	-17.27

71	0.30	31.75	29.75	-9.25
72	0.00	35	34.08	-2.97
73	0.52	19.75	15.67	-12.34
74	1.40	0	0	-33.29
75	0.82	29.25	24.5	-16.68
76	-0.18	31.25	21.25	-19.64
77	-0.18	36	35.58	0.89
78	0.16	30.5	29.08	-14.44
79	0.10	23.25	18.08	-22.97
80	0.30	17.75	14.33	-30.89
81	-0.18	32.75	32.25	-9.1
82	-0.18	25.25	8.75	-23.79
83	0.00	26.75	19.25	-29.96
84	0.16	27.25	19.75	-29.72
85	0.16	18.25	16.25	-22.19
86	-0.18	32	28.92	-18.12
87	0.00	35.5	35.33	1.08
88	0.00	24.5	26.08	-17.43
89	0.52	30	28.75	-10.75
90	1.52	1.75	2.83	-32.54
91	-0.08	36	35.25	-7.38
92	0.10	34.25	33.83	-3.3
93	0.16	26.5	19.92	-27.77

Abbreviations: FS, foveal sensitivity (dB); MS, macular sensitivity (dB); MD, mean deviation of HFA 10-2 (dB)
