# Supplementary material for: The qualitative assessment of optical coherence tomography and the central retinal sensitivity in patients with retinitis pigmentosa
Source: PLoS One. 2020 May 11;15(5):e0232700. doi: 10.1371/journal.pone.0232700 (PMC7213731; doi:10.1371/journal.pone.0232700)
Supplement: S3 Table — (PDF) [file pone.0232700.s005.pdf]

Table S3. Qualitative IS-OS Severity Grade (Graders' evaluation)

Patient's No.	Grader A	Grader B	Final Grade
1	4	4	4
2	1	1	1
3	4	4	4
4	5	5	5
5	5	5	5
6	5	5	5
7	2	2	2
8	5	5	5
9	4	4	4
10	4	4	4
11	3	3	3
12	4	4	4
13	2	2	2
14	2	2	2
15	5	5	5
16	4	4	4
17	4	4	4
18	3	4	4
19	1	2	2
20	1	2	2
21	3	3	3
22	1	2	2
23	3	3	3
24	2	2	2
25	1	2	2
26	1	1	1
27	2	2	2
28	5	4	4
29	2	2	2
30	5	5	5
31	4	4	4
32	4	3	3
33	3	2	2
34	5	5	5

35	2	2	2
36	5	5	5
37	4	4	4
38	4	3	3
39	4	4	4
40	5	5	5
41	1	1	1
42	4	4	4
43	3	3	3
44	4	4	4
45	2	2	2
46	3	3	3
47	4	4	4
48	3	4	4
49	3	4	4
50	2	2	2
51	3	4	4
52	2	2	2
53	2	2	2
5	4	4	4
55	1	1	1
56	1	1	1
57	5	5	5
58	1	1	1
59	1	1	1
60	1	2	2
61	3	3	3
62	1	1	1
63	2	2	2
64	4	4	4
65	2	2	2
66	2	2	2
67	2	3	3
68	1	1	1
69	3	3	3
70	3	2	2

71	2	2	2
72	1	1	1
73	2	3	3
74	3	4	4
75	2	2	2
76	2	2	2
77	1	1	1
78	2	2	2
79	2	2	2
80	3	3	3
81	2	2	2
82	2	2	2
83	2	3	3
84	4	3	3
85	3	3	3
86	2	2	2
87	1	2	2
88	2	2	2
89	1	2	2
90	5	5	5
91	1	1	1
92	1	1	1
93	3	3	3
